# Preferences for treatment of Attention-Deficit/Hyperactivity Disorder (ADHD): a discrete choice experiment

**DOI:** 10.1186/1472-6963-9-149

**Published:** 2009-08-13

**Authors:** Axel C Mühlbacher, Ina Rudolph, Hans-Joachim Lincke, Matthias Nübling

**Affiliations:** 1 GEB mbH - Empirical Consulting (Gesellschaft für Empirische Beratung), Postfach 1729, 79017 Freiburg, Germany; 2 IGM - Institut Gesundheitsökonomie und Medizinmanagement, Hochschule Neubrandenburg, Brodaer Straße 2, 17033 Neubrandenburg, Germany; 3 Janssen-Cilag GmbH, Johnsen&Johnsen Platz 1, 41470 Neuss, Germany

## Abstract

**Background:**

While there is an increasing emphasis on patient empowerment and shared decision-making, subjective values for attributes associated with their treatment still need to be measured and considered. This contribution seeks to define properties of an ideal drug treatment of individuals concerned with Attention-Deficit/Hyperactivity Disorder (ADHD). Because of the lack of information on patient needs in the decision-makers assessment of health services, the individuals' preferences often play a subordinate role at present. Discrete Choice Experiments offer strategies for eliciting subjective values and making them accessible for physicians and other health care professionals.

**Methods:**

The evidence comes from a Discrete Choice Experiments (DCE) performed in 2007. After reviewing the literature about preferences of ADHS we conducted a qualitative study with four focus groups consisting of five to eleven ADHS-patients each. In order to achieve content validity, we aimed at collecting all relevant factors for an ideal ADHS treatment. In a subsequent quantitative study phase (n = 219), data was collected in an online or paper-pencil self-completed questionnaire. It included sociodemographic data, health status and patients' preferences of therapy characteristics using direct measurement (23 items on a five-point Likert-scale) as well as a Discrete-Choice-Experiment (DCE, six factors in a fold-over design).

**Results:**

Those concerned were capable of clearly defining success criteria and expectations. In the direct assessment and the DCE, respondents attached special significance to the improvement of their social situation and emotional state (relative importance 40%). Another essential factor was the desire for drugs with a long-lasting effect over the day (relative importance 18%). Other criteria, such as flexibility and discretion, were less important to the respondents (6% and 9%, respectively).

**Conclusion:**

Results point out that ADHD patients and their family members have clear ideas of their needs. This is especially important against the backdrop of present discussions in the healthcare sector on the relevance of patient reported outcomes (PROs) and shared decision-making. The combination of the methods used in this study offer promising strategies to elicit subjective values and making them accessible for health care professionals in a manner that drives health choices.

## Background

This contribution seeks to measure needs and expectations of individuals concerned with Attention-Deficit/Hyperactivity Disorder (ADHD). Every approach to treatment is centred on the patient. It is therefore necessary to take adequate account of the patients' needs and values when providing and assessing treatments or technologies used in therapy. However, the assessment of health care professionals of the effects of illness and treatment on the patients' quality of life may differ from the way the patient feels about these. Differences between clinical findings - which are usually more or less objective - and the subjective experience of the patient give rise to many questions. Treatment is concerned not only with changing clinical symptoms, but furthermore with ensuring that patients themselves experience an improvement in their health state. When the efficacy of different treatments is viewed from the patients' perspective in this way, two "objectively" equal effective approaches to treatment can often be seen in very different lights. Moreover, it can be assumed that greater benefit from the patients' point of view will have a positive effect on compliance and also fundamentally strengthen the relationship between the patient and therapist. While it was shown in several open label studies, that drug treatment can improve the quality of life of ADHD patients and their families [1,2], it remains unknown which treatment aspects are perceived as most important.

At present, however, the assessment of medicinal products by physicians and other decision-makers is primarily based on the results of studies on clinical efficacy and safety. The patients' perspective and the needs and values of those concerned remain largely unknown and consequently play a rather subordinate role [3]. This is mainly due to the lack of adequate studies, as is the case with drug treatment in attention-deficit hyperactivity disorder (ADHD).

With a worldwide prevalence of 1 - 7%, ADHD is one of the most common psychiatric disorders in children and adolescents [4]. The core symptoms of ADHD consist in abnormal impulsive behaviour, a deficit in attention, and hyperactivity. They are relevant not only because of their prevalence, but also because of their consequences in the short, medium and long term, which far exceed the immediate concerns of the healthcare system [5]. In addition to family stress and problems, disturbed social behaviour, problems at school and at work and the attendant impaired quality of life, numerous significant long-term pathological developmental defects are known [6,7].

This condition is usually treated by using a multimodal therapeutic concept, consisting of a combination or series of different approaches to treatment that should be tailored to the individual needs of the patient. Standard approaches include psychosocial and behavioural, as well as psychopharmacological therapies [8]. Different drug treatments, in different pharmaceutical forms and with different therapeutic time frames, are available for patients. In Germany, the most frequently used drug is methylphenidate (MPH) [9]. The individual short- and long-acting MPH products have different effect profiles, which can be used to suit the individual everyday life, and thus have an additional effect on the quality of life of patients and their families [10].

The objective of this study is to define properties of an ideal ADHD drug treatment from the patient and parents' perspective. In addition to "classic" medical outcomes, other aspects important to the patient, e.g. quality of life and social behaviour, were included. A Discrete Choice Experiments (DCE) was performed in 2007 as a method to elicit preferences and making them accessible for physicians and other health care professionals.

## Methods

**Figure 1:**
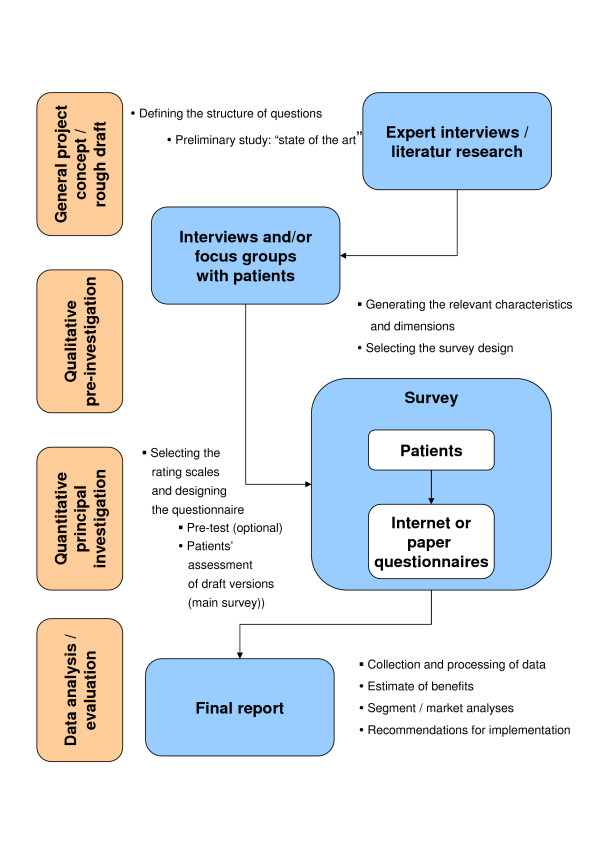
**Study design of the Discrete Choice Experiment**.

The study was divided into two parts: a qualitative part to collect relevant attributes and a quantitative main study to elicit the patients' preferences. The qualitative pre-investigation determined the desires and expectations of patients and their relatives with regard to the drug treatment of ADHD. In the principal investigation phase, these were then used as a basis for assessing the previously defined pools of characteristics with regard to their individual degree of relevance. The study is a social science survey. It does not contain personal data (completely anonymous survey), surgeries (tests, experiments, medication), biomedical research or additional data, like in many epidemiological investigations. Therefore an ethic vote was not necessary. Study goals were explained to all participants and all gave written informed consent for their participation. (Figure 1)

### Qualitative study

Prior to the main study, we performed a qualitative study to identify important aspects of an ideal ADHS treatment (summer 2007). Patient advocacy groups „ADHS Selbsthilfe e. V.“ asked patients to participate. They were interviewed in four focus groups (5 - 11 parents of patients). The aim was to ensure content validity, i.e. the accuracy of these characteristics. On the basis of literature research and the results of these focus groups 23 aspects were selected for the main study. In a pretest, the questionnaire was tested for comprehensibility by parents of patients and adolescent patients (n = 14). Based on the results of the pretest we finalised the questionnaire.

### Main study

The main study was performed as an anonymous survey, which started in early November 2007, using either online or paper questionnaires. Family members (mostly parents) and patients (>14 years) were contacted either in writing and distributing the paper-based questionnaire version with stamped addressed envelopes, or via email/internet. Patient advocacy groups helped in distributing paper-based questionnaires and the link to the online version. No personal data such as addresses, names or phone numbers were collected.

The questionnaire encompassed three main domains:

• Part A: Sociodemographic characteristics: e.g. age, gender, educational level, previous therapy, and member of patient advocacy group.

• Part B: Current health status (Health related Quality of Life (HRQoL), SF12v2, German version of the SOEP, SozioOekonomisches Panel), several questions concerning utilization of medical services.

• Part C: Assessment of importance of ADHS-therapy characteristics. Both methods are based on a multiattributive method to analyse preferences and assess combinations of characteristics.

• C1: Direct assessment of importance of 23 items derived from the qualitative pre-investigation. The importance of these criteria had to be assessed on a five-point Likert scale. For this direct measurement of preferences, respondents were directly asked for their subjective assessment of individual characteristics and dimensions.

• C2: Discrete Choice Experiment (DCE) used in this study included 6 factors: duration of effect, side effects, dosage, discretion, emotional state and social situation. For each of these factors, 2 exactly opposite (dichotomous) dimensions were given. In a fold-over design, 8 pairs of therapies were presented, each with 2 fictitious drugs (alternatives A and B). The contrary properties of the drugs were assigned at random. (Table 1)

**Table 1:** Example of a therapy pair

**Pair 1**	**Properties of treatment/therapy/Drug A**	**Properties of treatment/therapy/Drug B**
Duration of effect	Long (all day)	Short (half day)

Side effects	Loss of weight occurs	None

Dosage/dosage form	Always the same	Variable, combinable

Discretion	Intake of drug obvious	Intake of drug not obvious

Emotional state	Mood swings may occur	No mood swings

Social situation	No problems with friends, hobbies	Problems with friends, hobbies

Please tick:	∘ I would choose A	∘ I would choose B

In the original DCE no colours were used to indicate positive and negative attribute levels.

### Discrete Choice Experiment (DCE)

McFadden's work on discrete choice models [11] introduced feasible techniques for estimating a complete characteristics-based discrete choice model of demand. Attention to preferences as an input into health care decision-making is rooted in the application of decision theory to the understanding of personal choice [12]. The premise of the DCE is that rational individuals will always choose the alternative with the higher level of expected utility.

Discrete choice models have recently gained importance in the study of innovative health technologies and of non-market goods in the health care sector [13-15]. A key feature of these models is the specification of utilities associated with the alternatives in terms of choice characteristics and individual preferences [16].

Discrete Choice Experiments offer strategies for eliciting preferences to value health and health care [13]. The term patient preferences still lacks a consistent definition; despite these differences in definition, there appears to be convergence in the view that patient preferences are statements made by individuals regarding their needs, values and expectations and the relative importance of treatment properties. Therefore these preferences refer to the individual evaluation of dimensions of health outcomes. For measuring preferences in the healthcare sector, respondents are presented with 2 alternative (fictitious) health services/treatments, which combine different contrary product properties. The respondents have to select the alternatives in accordance with their individual preferences. The decision is based on a comparison of utility levels attained.

DCEs are limited to the use of only a few characteristics. It is very important to cover all the relevant fields when selecting the items for the DCE. The DCE used in this study included six dimensions: duration of effect, side effects, dosage, discretion, emotional state and social situation. All the 6 characteristics chosen were of high importance in the direct measurement and in the qualitative study. For each of these factors, two opposite (dichotomous) poles were given, e.g. 'long' and 'short' for duration of effect [17]. In a fold-over design, eight pairs of therapies were presented (labelled as alternatives A and B). The eight basic scenarios to be fold over were generated automatically as an orthogonal design using the software SPEED. The design demonstrated uncorrelated main effects and an efficiency of 100% [18].

The evaluation of the DCE involved descriptive methods (frequency tables, statistics of distribution), bivariate procedures (cross tables, comparisons of means, ANOVA), and complex multivariate procedures (factor analysis [PCA], probit models, logit models). The level of statistical significance in all analyses was p < 0.05 (2-sided).

## Results

### Patient Characteristics (Part A and B)

In November 2007, patients and parents completed the questionnaires (n = 219) in the 4-week documentation period. The vast majority of respondents were mothers of ADHD patients (79%); fathers (9%) and adolescent patients (6%). Most of the patients were male (83%), with a broad spectrum of ages (mean = 15 years). The following age groups were combined into one due to the small numbers of cases: up to 7 years, 19 - 25 years, and >25 years.

**Figure 2:**
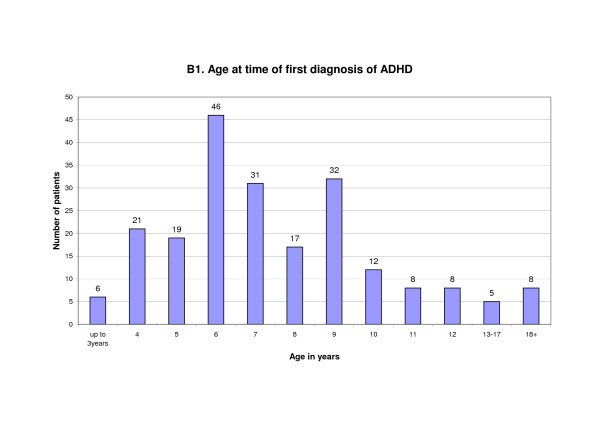
**Age at time of first diagnosis**.

The majority of patients had first been diagnosed with ADHD at an age of 6 - 9 years; most were 6 years old (range: 1 - 46 years; mean: 8.3 years). (Figure 2)

The first part of the questionnaire contained a total of 7 questions regarding the use of facilities available to parents: two-thirds (67%) of respondents belonged to a patient advocacy group at the time of the survey, 18% had previously been active in a patient advocacy group, and 15% had never belonged to such a group. A similar picture emerged for the use of psycho-educational facilities: 70% were using such facilities in the period of the survey, 17% had done so previously, and 14% had never made use of them. Much lower proportions had made use of parental training or coaching. Only 26% made use of parental training, and 22% preferred parental coaching. The vast majority had no experience of the following educational opportunities: 5% made use of systematic family therapy, 10% family assistance, and 8% therapeutic day centres.

**Figure 3:**
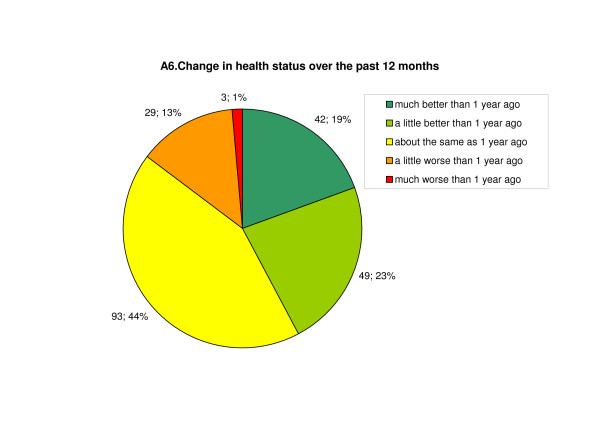
**Change in health status over the past 12 months**.

The health status of the child or adolescent concerned was classed as "very good" or "good" by 53% of respondents, "satisfactory" by 33%, "not so good" by 11%, and "bad" by 5%. With regard to changes in health status over the past 12 months, a marked improvement had been seen by 42%; 14% had noticed deterioration, and the remaining 44% had seen no change. (Figure 3)

The subjects were presented with a total of 10 treatment schemes to be assessed; information on their past experience was also relevant in this context. The vast majority of 91% professed to have had experience with drug therapy, and 76% were currently using it. About 50% had used ergo therapy and behavioural therapy before, while only 10% and 18%, respectively, were using them at the time of responding. About one-third (36%) had experience with therapy for the treatment of co-morbid disturbances. At the time of responding, 13% of subjects were still being treated for such problems.

**Figure 4:**
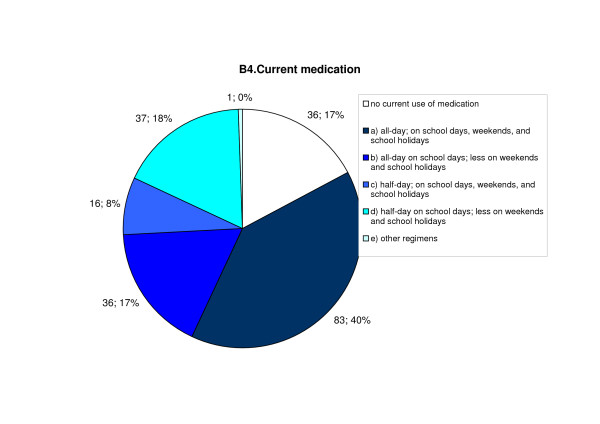
**Medication used during the study period**.

When asked about the current extent of their medication, 40% stated that they were using an "all-day drug treatment", on all days of the week. 17% used medication on school days, but less at weekends and in school holidays. 26% of respondents treated the disorder on a half-daily basis; 18% of these discontinued their treatment on weekends or during school holidays. A total of 17% professed to prefer non-drug methods. (Figure 4)

### Relevance of therapy characteristics: Direct assessment (Part C1)

We conducted a direct assessment in order to analyse the relevance of therapy characteristics. In part C1, respondents had to rate the importance of 23 therapy characteristics using a five-point Likert-Scale, ranging from "very important" to "not important". For subsequent evaluation, the ratings were transformed into a numerical range from 0 ("not important") to 100 ("very important"). It emerged that all but 2 of these items reached relatively high scores, meaning that patients consider most of them to be very important with regard to the quality of a therapy approach. This is not surprising, since only aspects were presented, that were rated as important according to the literature and the qualitative study/focus groups.

**Figure 5:**
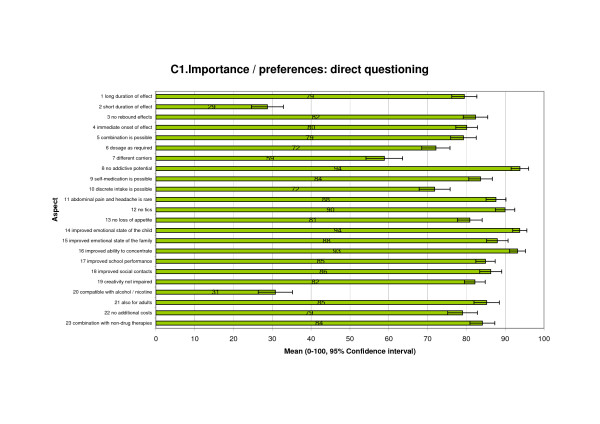
**Importance and preferences: direct questioning**.

The greatest relevance (100 - 90) was attributed to "improving the child's emotional state" (mean value = 94), "little or no addictive potential" (94), and "improved ability to concentrate" (93). (Figure 5)

Response styles are a source of contamination in questionnaire ratings [19]. Regarding the rating scale responses it should be stated that rating scales do not incorporate the trade-offs inherent in real-life decision-making. Therefore they threaten the validity of conclusions drawn from such research data. In order to draw valid conclusions we conducted a DCE.

### Preferences in the Discrete-Choice-Experiment (Part C2)

The DCE was built up with eight pairs (choices) each consisting of six dichotomous aspects. All the six characteristics chosen were of high importance in the direct measurement and in the qualitative study: duration of effect, side effects, dosage, discretion, emotional state, and social situation. To achieve maximum differentiation between the two alternatives a fold-over design was used: each of the eight pairs was presented to the subjects as alternatives A and B, with A being the exact "mirror image" of B.

This approach created varying decision options: some choices were relatively simple because one alternative was in almost all aspects apparently better than the other. On the contrary, in difficult decisions the advantages and disadvantages were mostly equally distributed which made the alternatives more equivalent and the choice more difficult.

From the patient's point of view, the 6 aspects presented influenced the choice of the best therapy to different degrees. The greatest importance was attributed to "enabling social contacts" (Item 6; 3,162 coefficient). This was followed by two items with almost the same degree of importance: by "emotional state: no mood swings" (Item 5; 1,644 coefficient) and "duration of effect: long (all day)" (Item 1; 1,437 coefficient). If one of these characteristics was present, this treatment alternative was very likely to be selected. These were followed at a considerable distance by "discretion" (Item 4; 0,727 coefficient), "dosage" (Item 3; 0,468 coefficient), and "side effects" (Item 2; 0,470 coefficient). A supplementary (partial) log-likelihood analysis as proposed by Lancsar et al. [15] yielded to the same hierarchy as the interpretation based on the six item-coefficients. All 6 aspects were statistically significant, with a level of p < 0.001 for Items 1, 4, 5 and 6, and p < 0.01 for Items 2 and 3. (Table 2)

**Table 2:** Results of the random effects model (DCE)

**Item**	**Characteristic and dimension**	**Coefficient**	**SE (coeff)**	**Significance**	**Partial log likelihood when item omitted**
1	Duration of effect: long (all day)	1.437	0.169	***	-797.3
	Duration of effect: short (half day)	-1.437	0.169	***	

2	Side effects: none	0.470	0.147	**	-756.2
	Side effects: loss of weight may occur	-0.470	0.147	**	

3	Dosage/dosage form: variable, combinable	0.468	0.155	**	-755.3
	Fixed dosage and dosage form	-0.468	0.155	**	

4	Discretion: intake not obvious	0.727	0.143	***	-763.7
	Discretion: intake obvious	-0.727	0.143	***	

5	Emotional state: no mood swings	1.644	0.167	***	-813.6
	Emotional state: mood swings may occur	-1.644	0.167	***	

6	Social situation: no problems with friends, hobbies	3.162	0.169	***	-1055.1
	Social situation: problems with friends, hobbies	-3.162	0.169	***	

	Model constant	-4.144	0.294	***	

***: p < 0.001, **: p < 0.01, *: p < 0.05

Model parameters:

Wald Chi^2 ^(df = 6) = 379.67

Log likelihood = -750.8

Prob > Chi^2 ^= 0.0000 (i.e. p < 0.001, ***)

Prob >= Chibar^2 ^= 0.495

At the end of the questionnaire, subjects were asked about their degree of satisfaction with their present treatment. In total, 58% were "satisfied" or "very satisfied", while about one-third chose the category "yes and no", and 11% expressed a negative opinion of their present therapy.

## Discussion

The objective of the present study was to elicit the preferences of patients and their parents with regard to the drug treatment of ADHD. Based on a qualitative (pre) investigation to ensure content validity, quantitative measurements of preference were subsequently performed, including a Discrete Choice Experiment (DCE).

By means of multiattributive preference measurements, DCE designs can provide valid and robust results on relevant outcomes of ADHD treatment from the patient's point of view.

With a combination of direct questions - i.e. direct measurements of importance - and a DCE, it is possible to simulate real-life decision-making situations for patients. The combination of direct assessment of importance and DCE is a valid combined survey technique for eliciting preferences of patients with ADHS. The former ensures content validity (the possibility to measure a longer list of potentially important aspects), the latter has the advantage to combine positive and negative therapy characteristics and to avoid the problem of ceiling effects and "all-is-important" results.

There are a few limitations to this study that need to be pointed out. First, for practicability DCE needs to be performed with preferably the least number of parameter and pairs. The level of complexity of our study turned out to manageable for the participants. Second, there is the difficulty that the questionnaire was widely spread (paper and pencil version and online version). The number of patients who got into contact with the questionnaire is unknown - therefore, response rates cannot be calculated. The intense usage of the paper version shows that offering the paper copy is important and useful - at least in this patient population. Third, the study participants are probably more committed and well informed than the average of ADHS patient, because most of the respondents were therapy-experienced and members of patient advocacy groups. We do not know if this selection could have potentially biased the preference assessments.

In spite of these limitations, however, two results stand out. Against the backdrop of ADHD, multiattributive procedures allow an assessment of the benefit of therapy from the point of view of patients and parents. Therefore, from a methodological point of view, both approaches should always be used in parallel for preference studies, as a direct measurement of importance makes it possible to cover more aspects than can be elicited in combination by the DCE. The results of the two methods - the direct and indirect (DCE) method - are largely equivalent: as can be seen from the results of this study, patients and parents have clear ideas about their own needs, and are able to assess different therapeutic approaches. Patients or their parents attach particular importance to an improvement of their social situation and emotional state. This means that a balanced mood and a positive social environment (friends and hobbies) contribute significantly to the overall patient benefit from a drug treatment. It can be concluded that these treatment outcomes heavily influence parents' or older patients' choice to administer a drug treatment. A long duration of effect throughout the day is considered almost as desirable as the improvement of the emotional state, whereas other factors, such as discretion and flexibility, appear to be less important to patients.

## Conclusion

The patients' view and desires in healthcare decisions (e.g. attributes of therapies) are often not sufficiently considered. The Institute of Medicine report in 2001, "Crossing the Quality Chasm" [20] emphasizes that health decisions should be customized based on patients' needs and values. However, in times of limited healthcare resources, shared decision making as patients involvement in treatment decisions have been encouraged in recent years. This requires an understanding of patients' priorities concerning treatment decision-making. These findings supply important information on the benefit of therapy from the patients' point of view, and should be increasingly taken into account when deciding on therapeutic approaches. They make an important contribution to the patient-oriented optimization of treatments and can thus help to improve the final outcome of treatment. If patient needs are taken into account adequately, it should be safe to assume that this will increase compliance, which is an essential requirement. This study also supports the efforts for increased consideration of patient benefit as a new quality criterion in the assessment of drug treatments. Especially where clear differentiation between treatments in terms of medical and financial aspects is difficult, comprehensive information on patient benefits to be expected can be very useful in the prioritization of treatment approaches. Studies of this type can thus help to stimulate new discussions in the healthcare sector, leading to the formulation of increasingly patient-centred care concepts in the long term.

It would also be useful to conduct further studies that, in addition to assessing the preferences of patients and parents, will consider the choices and decisions of actual and potential decision-makers in the healthcare sector. In this case, the comparison of patient preferences and expert opinions may reveal discrepant aspects of treatment that need improvement. A resource-oriented, balanced approach to treatment, which also serves the needs of the individual patient, will not be possible unless full account is taken of the patients' actual demands.

## Competing interests

Janssen-Cilag GmbH Germany granted the research funding. IR is in a working relationship with Janssen Cilag GmbH Neuss, Germany; Janssen Cilag GmbH manufacturers medication for ADHD.

## Authors' contributions

ACM and MN developed the design of the study, data collection and performed the statistical analysis, and interpretation of data. All authors contributed to interpretation of the data and the critical revision of the manuscript, read and approved the final manuscript.

## Pre-publication history

The pre-publication history for this paper can be accessed here:


